# Toward nonparametric diffusion‐$T_{1}$ characterization of crossing fibers in the human brain

**DOI:** 10.1002/mrm.28604

**Published:** 2020-12-10

**Authors:** Alexis Reymbaut, Jeffrey Critchley, Giuliana Durighel, Tim Sprenger, Michael Sughrue, Karin Bryskhe, Daniel Topgaard

**Affiliations:** ^1^ Department of Physical Chemistry Lund University Lund Sweden; ^2^ Random Walk Imaging AB Lund Sweden; ^3^ Spectrum Medical Imaging Sydney Australia; ^4^ Karolinska Institute Stockholm Sweden; ^5^ GE Healthcare Stockholm Sweden; ^6^ Charlie Teo Foundation Sydney Australia

**Keywords:** diffusion‐relaxation correlation, fiber‐specific microstructure, inverse Laplace transform, multivariate distribution, orientation distribution function, tensor‐valued diffusion encoding

## Abstract

**Purpose:**

To estimate $T_{1}$ for each distinct fiber population within voxels containing multiple brain tissue types.

**Methods:**

A diffusion‐$T_{1}$ correlation experiment was carried out in an in vivo human brain using tensor‐valued diffusion encoding and multiple repetition times. The acquired data were inverted using a Monte Carlo algorithm that retrieves nonparametric distributions $P(D,R_{1})$ of diffusion tensors and longitudinal relaxation rates $R_{1}\;=\;1/T_{1}$. Orientation distribution functions (ODFs) of the highly anisotropic components of $P(D,R_{1})$ were defined to visualize orientation‐specific diffusion‐relaxation properties. Finally, Monte Carlo density‐peak clustering (MC‐DPC) was performed to quantify fiber‐specific features and investigate microstructural differences between white matter fiber bundles.

**Results:**

Parameter maps corresponding to $P(D,\;R_{1})$’s statistical descriptors were obtained, exhibiting the expected $R_{1}$ contrast between brain tissue types. Our ODFs recovered local orientations consistent with the known anatomy and indicated differences in $R_{1}$ between major crossing fiber bundles. These differences, confirmed by MC‐DPC, were in qualitative agreement with previous model‐based works but seem biased by the limitations of our current experimental setup.

**Conclusions:**

Our Monte Carlo framework enables the nonparametric estimation of fiber‐specific diffusion‐$T_{1}$ features, thereby showing potential for characterizing developmental or pathological changes in $T_{1}$ within a given fiber bundle, and for investigating interbundle $T_{1}$ differences.

## 1 INTRODUCTION

While diffusion MRI^1^, ^2^, ^3^, ^4^, ^5^ has provided enhanced sensitivity to tissue microstructure in vivo by capturing the translational motion of water molecules, diffusion‐relaxation MRI (drMRI)^6^, ^7^, ^8^, ^9^, ^10^, ^11^, ^12^, ^13^, ^14^ additionally reports on the local chemical composition of the aqueous phase. For instance, the longitudinal relaxation time $T_{1}$ is mainly determined in vivo by cross relaxation, magnetization transfer, and spin diffusion with macromolecules in general,^15^, ^16^, ^17^ by myelin lipids in particular,^18^, ^19^, ^20^, ^21^, ^22^ and by the interplay between relaxation and diffusion.^23^ However, microstructural studies have been hindered by the fact that the measured drMRI signal is only sensitive to the voxel‐averaged diffusion‐relaxation profile, with typical cubic‐millimeter voxels comprising multiple cell types and the extracellular space.^24^, ^25^, ^26^, ^27^, ^28^

Three strategies were explored to alleviate the lack of specificity of the drMRI signal. First, multiple models and signal representations have been developed to relate either the diffusion‐$T_{2}$ ^29^, ^30^, ^31^, ^32^ or diffusion‐$T_{1}$ ^7^, ^8^, ^33^ MRI signal to the voxel content. However, these approaches rely on compartmental/functional assumptions and/or on model‐selection strategies that may disagree with the underlying tissue microstructure.^34^, ^35^, ^36^ Second, while nonparametric inversion of the diffusion‐relaxation NMR signal is already common practice in the porous media field,^37^, ^38^, ^39^, ^40^ nonparametric inversion techniques of the drMRI signal have also been developed,^14^, ^41^, ^42^ with applications ranging from porous media^6^ to biological tissues such as the heart,^43^ spinal cord,^44^, ^45^ placenta,^11^ and brain.^46^ However, these techniques have so far only been employed to retrieve 2D or 3D diffusivity–relaxation distributions.^14^, ^42^ Their lack of specificity in terms of diffusion orientation thus render them unable to isolate subparts of the distribution belonging to distinct subvoxel anisotropic diffusion environments, for example, white matter (WM) fiber populations. Third, “tensor‐valued” diffusion‐encoding gradient waveforms have enhanced the specificity of the data itself by targeting specific features of the intravoxel diffusion profile^47^, ^48^, ^49^, ^50^, ^51^, ^52^, ^53^, ^54^ *via* an axisymmetric encoding tensor b introduced in Refs. [3, 55, 56]. Tensor‐valued diffusion acquisition schemes have since been used to further investigate signal representations^50^, ^57^, ^58^, ^59^ and models.^32^, ^60^, ^61^, ^62^, ^63^, ^64^

The advent of tensor‐valued diffusion measurements has resulted in the development of nonparametric Monte Carlo signal inversion algorithms of the 2D diffusion^65^ and 6D diffusion‐$T_{1}$‐$T_{2}$ ^12^ NMR signals in porous media, and of the 4D diffusion^66^ and 5D diffusion‐$T_{2}$ ^13^ MRI signals in the in vivo brain. Although noise sensitive,^36^ these algorithms do not rely on any compartmental/functional assumption regarding the voxel content, nor on any regularization^67^, ^68^, ^69^, ^70^ narrowing the space of suitable solutions to the inverse problem. They are also not limited by constraints regarding data compression, or restricted to dense acquisition sampling schemes that are difficult to extend to higher dimensions, unlike previous works in Ref. [71] and Refs. [41, 45, 72, 73, 74], respectively. Enhanced by methods aiming to visualize and quantify fiber‐specific properties, Monte Carlo signal inversions have recently provided critical sensitivity and specificity to fiber‐specific $T_{2}$ values.^75^, ^76^, ^77^ However, this work has yet to be extended to fiber‐specific $T_{1}$‐values, which are of particular interest to evaluate changes in bundle‐specific myelin contents^78^ relevant to the study of neurodevelopment, plasticity, aging, and neurological disorders.^79^, ^80^, ^81^

In this work, we leverage nonparametric distributions $P(D,R_{1})$ of diffusion tensors D"/> and longitudinal relaxation rates $R_{1}\;=\;1/T_{1}$ obtained *via* Monte Carlo inversion of a 5D diffusion‐$T_{1}$ weighted in vivo human brain dataset to resolve subvoxel diffusion‐$R_{1}$ components. We first estimate parameter maps of $P(D,\;R_{1})$’s statistical descriptors and extract orientation‐resolved $T_{1}$ values within the pool of highly anisotropic components output by the Monte Carlo inversion algorithm. These $T_{1}$ values are then color‐mapped onto nonparametric orientation distribution functions (ODFs)^75^, ^76^ and quantified in terms of median value and precision using Monte Carlo density‐peak clustering (MC‐DPC).^77^ In particular, we identify significant differences with respect to $T_{1}$ relaxation between major WM bundles without relying on limiting assumptions, albeit in a single healthy volunteer.

After describing how our in vivo human brain data was acquired in Section 2.1, we lay down the theory underlying the Monte Carlo inversion algorithm and the statistical descriptors of $P(D,R_{1})$ in Section 2.2, and detail our ODF and MC‐DPC procedures in Section 2.3. We then present our results in Section 3 and discuss them in Section 4, and conclude in Section 5.

## 2 METHODS

### 2.1 In vivo human brain data

**FIGURE 1:**
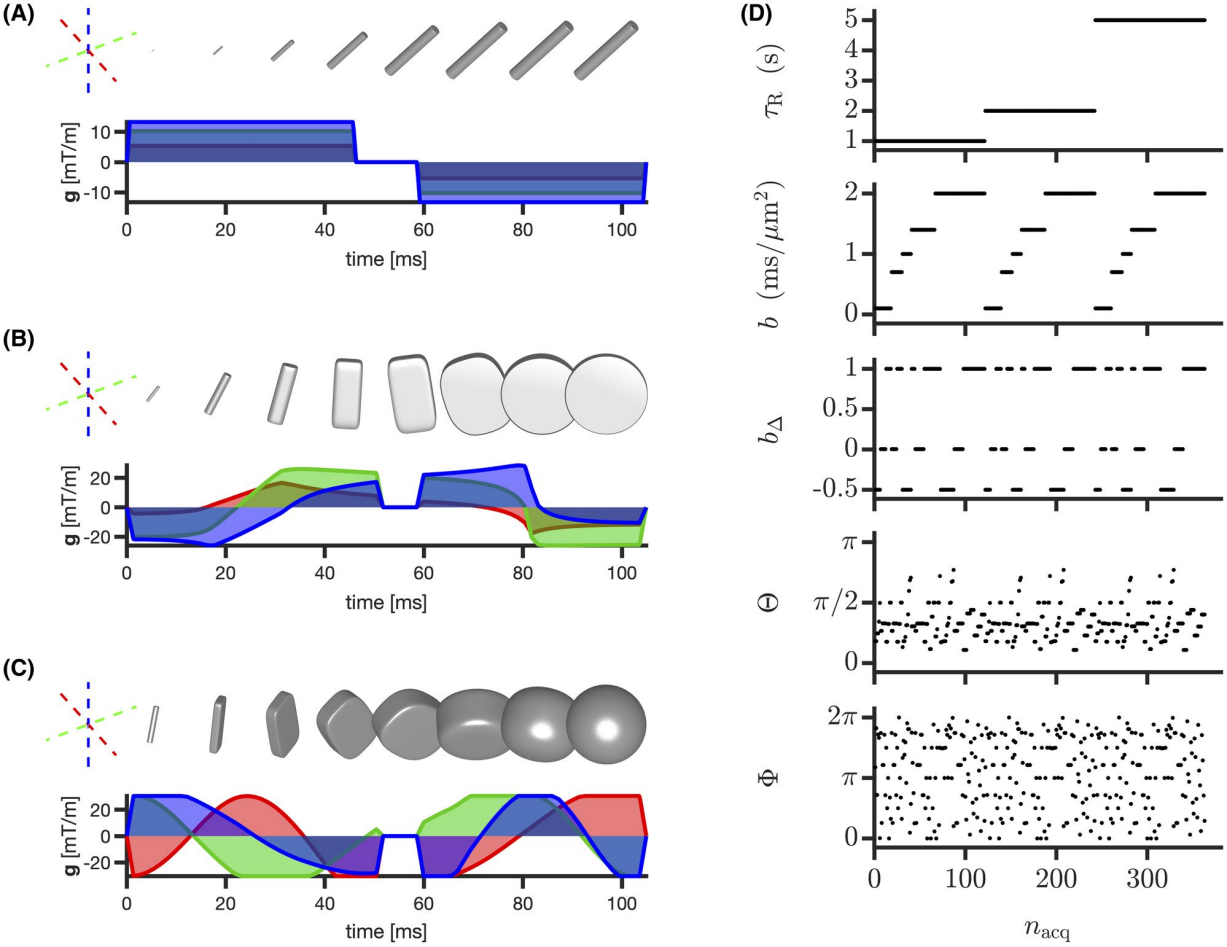
Visualization of our acquisition scheme. (A, B, and C) Gradient waveforms **g**(*t*) yielding linear, planar, and spherical diffusion encoding, respectively. Each color relates each gradient component to a given orthogonal axis of the spatial frame of reference. Tensor glyphs represent the b‐tensor progressively acquiring its final size (trace) b, shape (normalized anisotropy) $b_{\Delta }\in \left[‐0.5,\;1\right]$ and orientation (Θ,Φ) at the end of a given waveform.^49^, ^54^ D, Acquisition parameters as a function of sorted acquisition point index $n_{\mathrm{acq}}$. $\tau_{R}$ denotes the varying repetition time of our acquisition scheme

Data collection was approved by the Spectrum Medical Imaging local ethics committee. A healthy volunteer was scanned on a 3T GE 750w equipped with a 32‐channel receiver head and neck GEM coils (only 12‐16 channels used for head) using a prototype GE multidimensional diffusion (MDD) spin‐echo sequence with EPI readout, echo time $\tau_{E}\;=\;120$ ms, FOV = 240 × 240 × 12 ${\mathrm{mm}}^{3}$, voxel size = 3 × 3 × 3 ${\mathrm{mm}}^{3}$, fat saturation pulses,^82^ and ASSET acceleration factor=2, customized for tensor‐valued diffusion encoding,^57^, ^83^ and variable repetition time $\tau_{R}$. Tensor‐valued diffusion encoding was performed with numerically optimized^84^ Maxwell‐compensated^85^ waveforms whose gradient power spectra share similar frequency contents.^86^, ^87^, ^88^ The same tensor‐valued diffusion‐weighted sequence, including linear, planar, and spherical b‐tensors with a maximal b‐value of $2\;\text{ms}/\mu m^{2}$, was repeated for $\tau_{R}\;=\;1$, 2 and 5 seconds. The five dimensions of the resulting 20‐minute 363‐point acquisition scheme, shown in Figure 1, encode information allowing estimation of the 5D distribution $P(D,R_{1})$. The signal‐to‐noise ratio (SNR) of this dataset, estimated across voxels of the corona radiata by computing the mean‐to‐standard‐deviation ratio of the spherically encoded diffusion signal at *b* = 0.1 ms/$\mu m^{2}$ and $\tau_{R}\;=\;5$ seconds (see Supporting Information of Ref. [83]), equals 40. As indicated above, only 4 axial slices were acquired in order to limit the acquisition time. While an inversion‐recovery slice‐shuffling sequence could drastically reduce acquisition time,^9^, ^10^ our prototype sequence is currently limited to sequential slices and lacks eddy current nulling.^89^

### 2.2 Nonparametric Monte Carlo inversion

#### 2.2.1 Signal fitting and bootstrapping

We modified the 5D Monte Carlo inversion algorithm found in Ref. [13] and pioneered by Ref. [37] to analyze the diffusion‐$T_{1}$ dataset described in Section 2.1. Let us consider axisymmetric diffusion tensors, parameterized by their axial diffusivity $D_{\|}$, radial diffusivity $D_{⊥}$, and orientation (θ,ϕ). An alternative parameterization includes the isotropic diffusivity $D_{\mathrm{iso}}\;=\;\left(D_{\|}+2D_{⊥}\right)/3$ and normalized anisotropy $D_{\Delta }\;=\;\left(D_{\|}‐D_{⊥}\right)/\left(D_{\|}+2D_{⊥}\right)\in \left[‐0.5,1\right]$.^49^, ^54^, ^90^, ^91^ Our Monte Carlo inversion technique retrieves nonparametric intravoxel 5D distributions $P\left(D,R_{1}\right)\equiv P\left(D_{\|},\;D_{⊥},\;\theta ,\;\phi ,\;R_{1}\right)$ by fitting diffusion‐$T_{1}$ weighted signals with a weighted sum of N components $\left(D_{n},\;R_{1,n}\right)\equiv \left(D_{\|,n},\;D_{⊥,n},\;\theta_{n},\;\phi_{n},\;R_{1,n}\right)$, with 1≤n≤N. Given that the $T_{1}$‐weighting of the dataset detailed in Section 2.1 is provided through a spin‐echo sequence with constant echo time $\tau_{E}$ and variable repetition time $\tau_{R}$, the algorithm inverts the following discretized signal equation^92^:

(1) $$S_{m}\;\;=\;\;\sum_{n\;=\;1}^{N}w_{n}\;\exp(‐b_{m}:D_{n})\left[1‐2\exp([\tau_{E}/2‐\tau_{R,m}]R_{1,n})+\exp(‐\tau_{R,m}R_{1,n})\right],$$

where $S_{m}$ is the *m*th acquired signal, $w_{n}$ is the weight of the nth component (normalized so that $\sum_{n=1}^{N}w_{n}=S_{0}=S\left(b=0,\tau_{R}\to +\infty \right)$), b is the diffusion‐encoding tensor (b‐tensor),^47^, ^48^, ^49^, ^50^, ^51^, ^54^ and “:” is the Frobenius inner product. The Monte Carlo inversion algorithm randomly samples components $\left(D_{\|,n},D_{⊥,n},\theta_{n},\phi_{n},R_{1,n}\right)$ within the following ranges, $D_{\|},D_{⊥}\in \left[5\times 10^{‐3},5\right]\;\mu m^{2}/\mathrm{ms}$, $\cos\left(\theta \right)\in [0,1)$, ϕ∈[0,2π) and $R_{1}\in \left[0.1,\;2\right]\;s^{‐1}$, and estimates the weights $w_{n}$ quantifying the components’ propensity to fit the acquired signals *via* non‐negative least‐squares fitting ^69^, ^70^, ^71^, ^93^, ^94^ of Equation (1). This process is repeated iteratively following a quasi‐genetic filtering detailed in Refs. [12, 13, 65, 95]. Using the same wording as these references, we used $N_{\mathrm{in}}\;=\;200$ initial components, $N_{p}\;=\;30$ proliferation steps, $N_{m}\;=\;30$ mutation/extinction steps, and $N_{\mathrm{out}}\;=\;50$ output components. Embracing the inherent ill‐conditioning of Laplace inversion problems, we performed bootstrapping with replacement^96^, ^97^ on the data and estimated for each voxel an ensemble of $N_{b}\;=\;96$ plausible sets of components, also called “bootstrap solutions,” each denoted by ${\left\{\left(D_{\|,n},\;D_{⊥,n},\;\theta_{n},\;\phi_{n},\;R_{1,n},\;w_{n}\right)\right\}}_{1\leq n\leq N\;=\;50}$.

#### 2.2.2 Statistical descriptors and binning

The final solution of the Monte Carlo inversion algorithm, $P(D,R_{1})$, can be understood as the median of all bootstrap solutions. Following previous works,^13^, ^36^ we quantified the main features of this final solution by computing the median across bootstrap solutions of the per‐bootstrap means ${\text{Med}}_{\left(n_{b}\right)}\;\left(E{\left[\chi \right]}_{n_{b}}\right)$, variances ${\text{Med}}_{\left(n_{b}\right)}\;(V{\left[\chi \right]}_{n_{b}})$, and covariances ${\text{Med}}_{\left(n_{b}\right)}\;(C{\left[\chi ,\chi^{\prime }\right]}_{n_{b}})$ of $\chi ,\chi^{\prime }\;=\;D_{\mathrm{iso}},D_{\Delta }^{2},R_{1}$, with $1\leq n_{b}\leq N_{b}\;=\;96$. The median operator ${\text{Med}}_{\left(n_{b}\right)}\left(\;\cdot \;\right)$ acts across bootstrap solutions, and $E{\left[\;\cdot \;\right]}_{n_{b}}$, $V{\left[\;\cdot \;\right]}_{n_{b}}$ and $C{\left[\;\cdot ,\cdot \;\right]}_{n_{b}}$ denote the per‐bootstrap average, variance, and covariance over bootstrap solution $n_{b}$, respectively. For simplicity, we implicitly omit the median operator when addressing a statistical descriptor, thereby writing averages, variances, and covariances as “E[χ],” “V[χ],” and “$C[\chi ,\chi^{\prime }]$,” respectively.

**FIGURE 2:**
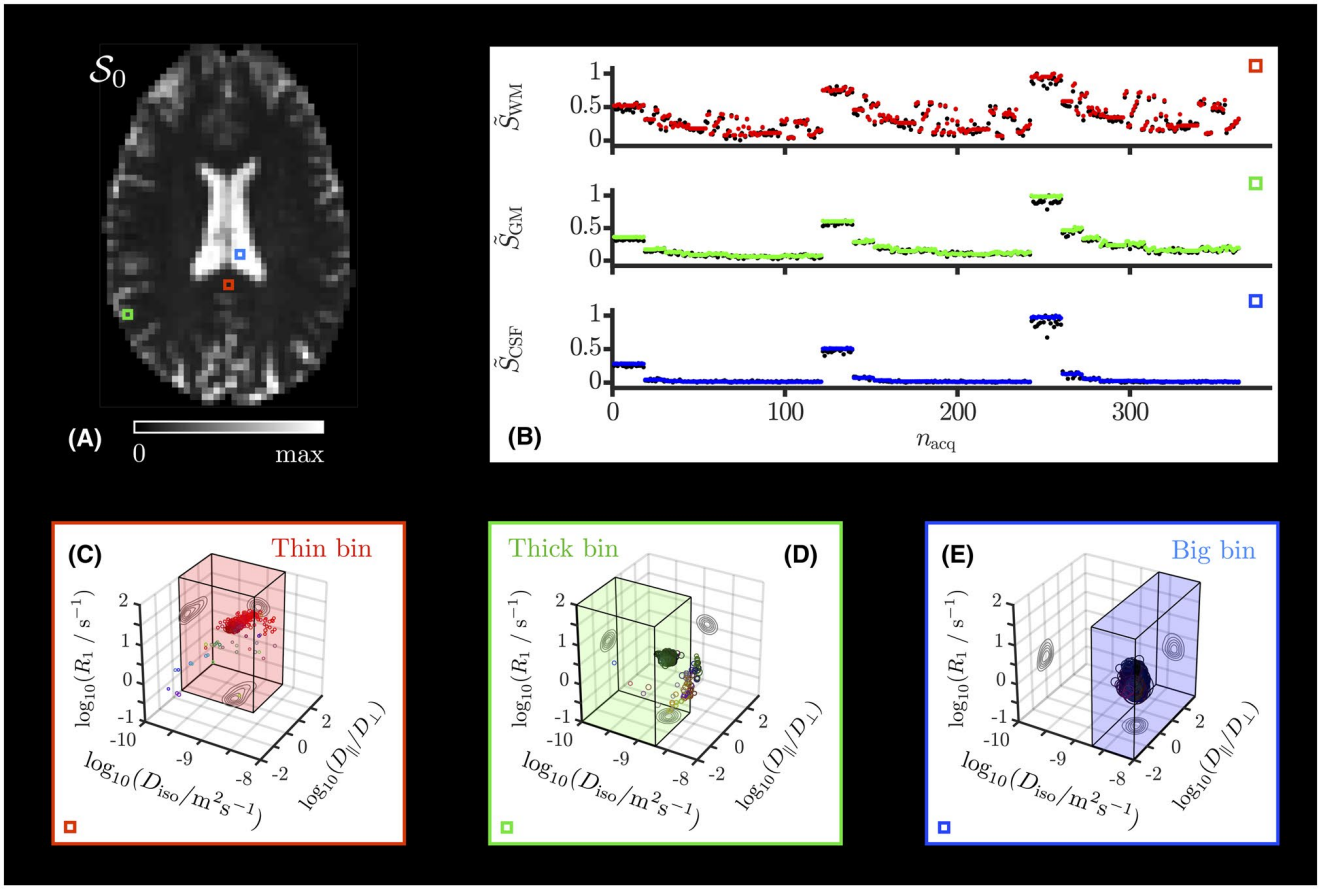
Monte Carlo fitted signal and retrieved 5D distributions $P(D,R_{1})$ in typical voxels. A, $S_{0}$ map estimated by the Monte Carlo inversion. The colored squares delineate typical WM (red), GM (green), and CSF (blue) voxels. B, Normalized signal $\tilde{S}\;\;=\;\;S/\max\left(S\right)$ measured (black points) and fitted (colored points) in the archetypal voxels of panel A as a function of the sorted acquisition point index $n_{\mathrm{acq}}$ of Figure 1. (C, D, E) Nonparametric distributions $P(D,R_{1})$ estimated for the archetypal voxels of panel A and reported as scatter plots in a 3D space of the logarithms of the longitudinal relaxation rate $R_{1}$, isotropic diffusivity $D_{\mathrm{iso}}$, and axial‐radial diffusivity ratio $D_{\|}/D_{⊥}$. Diffusion orientations (θ,ϕ) are color‐coded according to $\left[\text{red, green, blue}\right]=\left[\sin\;\theta \;\cos\;\phi ,\;\sin\;\theta \;\sin\;\phi ,\;\cos\;\theta \right]\;\times \left|D_{\left|\right|}‐D_{⊥}\right|/\max\left(D_{\left|\right|},D_{⊥}\right)$. Symbol area is proportional to the statistical weight $w_{n}/S_{0}$ of the corresponding component **n**. The contour lines on the sides of the plots represent projections of the 5D distributions $P(D,R_{1})$ onto the respective 2D planes. The “thin,” “thick,” and “big” bins defined in Section 2.2.2 are illustrated as colored boxes in the panels where they are most relevant. The colors bounding panels C, D, and E match those of the highlighted voxels in panel A

Tissue‐specific statistical descriptors can be extracted by subdividing the 5D configuration space of $P(D,R_{1})$ into multiple bins. For instance, the “thin,” “thick,” and “big” bins introduced in Refs. [13, 54] aim to isolate the signal contributions from WM, gray matter (GM), and cerebrospinal fluid (CSF), respectively. The bin boundaries, illustrated in the panels C, D, and E of Figure 2, were defined as follows:

- “thin” bin within $D_{\mathrm{iso}}\in \left[0.1,\;2\right]\;\mu m^{2}/\mathrm{ms}$, $D_{\left|\right|},D_{⊥}\in \left[4,1000\right]$ and $R_{1}\in \left[0.01,\;10\right]\;s^{‐1}$.
- “thick” bin within $D_{\mathrm{iso}}\in \left[0.1,\;2\right]\;\mu m^{2}/\mathrm{ms}$, $D_{\|}/D_{⊥}\in \left[0.01,\;4\right]$ and $R_{1}\in \left[0.01,\;10\right]\;s^{‐1}$.
- “big” bin within $D_{\mathrm{iso}}\in \left[2,\;10\right]\;\mu m^{2}/\mathrm{ms}$, $D_{\|}/D_{⊥}\in \left[0.01,\;1000\right]$ and $R_{1}\in \left[0.01,\;10\right]\;s^{‐1}$.

Bin‐specific statistical descriptors were estimated following the above process for the retrieved components specifically falling into each bin. Note that this manual binning consists merely in a preliminary attempt to comprehend the rich information contained in $P(D,\;R_{1})$, and is as such a limitation that could be mitigated by automatic clustering methods or by higher dimensional versions of data‐driven techniques such as those of Refs. [46, 98].

### 2.3 Orientation distribution functions and Monte Carlo density‐peak clustering

Orientation distribution functions were defined from the thin‐bin components output by the Monte Carlo inversion of Section 2.2.1 using the procedure detailed in Refs. [75, 76, 95]. This procedure consists in mapping the set of thin‐bin components onto the nodes of a dense spherical mesh $\left\{\left(\theta_{\mathrm{mesh}},\phi_{\mathrm{mesh}}\right)\right\}$, building up the ODF radii from the thin‐bin component weights w and orientations (θ,ϕ). In addition, it enables to compute per‐bootstrap orientation‐specific diffusion‐relaxation means $\hat{E}{\left[\chi \right]}_{n_{b}}\left(\theta_{\mathrm{mesh}},\;\phi_{\mathrm{mesh}}\right)$, with $\chi \equiv D_{\mathrm{iso}},D_{\Delta }^{2},R_{1}$. Besides coloring ODFs according to local orientation, this mapping allows to color ODFs according to the local value ${\mathrm{Med}}_{\left(n_{b}\right)}\left(\hat{E}{\left[\chi \right]}_{n_{b}}\left(\theta_{\mathrm{mesh}},\;\phi_{\mathrm{mesh}}\right)\right)$, which we used to visualize orientation‐specific diffusion‐relaxation quantities. For simplicity, the short‐hand notation “$\hat{E}\left[\chi \right]$” is now retained for ${\mathrm{Med}}_{\left(n_{b}\right)}\left(\hat{E}{\left[\chi \right]}_{n_{b}}\left(\theta_{\mathrm{mesh}},\;\phi_{\mathrm{mesh}}\right)\right)$.

MC‐DPC,^77^ that is, a combination of the Monte Carlo inversion algorithm of Section 2.2.1 and density‐peak clustering,^99^ was used to quantify the median value and precision of orientation‐resolved means of $\chi \equiv D_{\mathrm{iso}},\;D_{\Delta }^{2},\;R_{1},\;T_{1}\;=\;1/R_{1}$ across bootstrap solutions, which we employed to detect differences between subvoxel fiber populations. Briefly, MC‐DPC first delineates subvoxel clusters as orientational aggregates of thin‐bin solutions, which are interpreted as orientational regions of interest associated with subvoxel fiber populations. MC‐DPC then computes per‐bootstrap cluster‐specific (orientation‐resolved) means $\overset{{}^{\circ}}{E}{\left[\chi \right]}_{n_{b},n_{c}}$, where $n_{c}$ denotes the cluster index. Finally, one extracts cluster‐specific medians and interquartile ranges of $\overset{{}^{\circ}}{E}{\left[\chi \right]}_{n_{b},n_{c}}$ across bootstrap solutions. For simplicity, the short‐hand notation “$\overset{{}^{\circ}}{E}[\chi ]$” is now used to describe the collection of orientation‐resolved means $\overset{{}^{\circ}}{E}{\left[\chi \right]}_{n_{b},n_{c}}$ originating from all bootstrap solutions $n_{b}$ and all clusters $n_{c}$. Also, $\overset{{}^{\circ}}{E}[T_{1}]$ and $\overset{{}^{\circ}}{E}[R_{1}]$ were computed separately, as both quantities are commonly found in the MRI literature and $\overset{{}^{\circ}}{E}[T_{1}]$ does not generally equal $1/\overset{{}^{\circ}}{E}[R_{1}]$.

The ODF and MC‐DPC procedures are detailed for the diffusion‐$T_{1}$ case in Supporting Information. In addition, an in silico evaluation of these techniques is provided in Supporting Information Figures S1 and S2, demonstrating their accuracy in capturing relaxation‐based differences across distinct subvoxel fiber populations at intermediate‐to‐high SNR levels.

## 3 RESULTS

**FIGURE 3:**
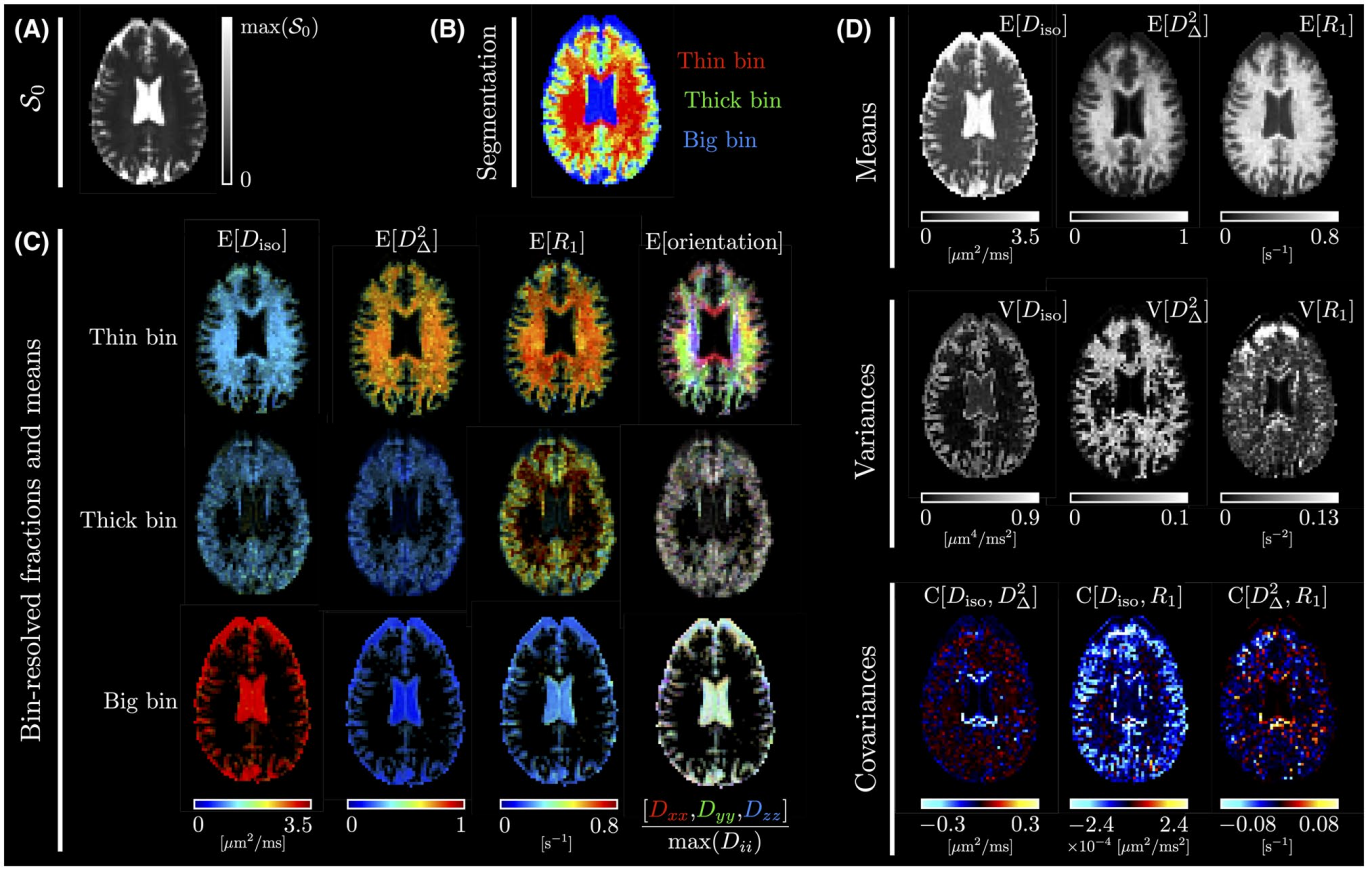
Typical axial maps of the statistical descriptors described in Section 2.2.2. While panel (A) shows the non‐diffusion‐weighted and non‐$R_{1}$‐weighted signal $S_{0}$, panel (B) presents a segmentation map of the brain into the thin, thick, and big bins defined in Section 2.2.2, colored according to $\left[\text{red, green, blue}\right]\;\;=\;\;\left[f_{\mathrm{thin}},f_{\mathrm{thick}},f_{\mathrm{big}}\right]/\text{max}\left(f_{\mathrm{thin}},f_{\mathrm{thick}},f_{\mathrm{big}}\right)$. Panel (C) features maps of the bin‐specific means of $D_{\mathrm{iso}}$, $D_{\Delta }^{2}$, $R_{1}$ and orientation. The bin‐specific averaged subvoxel orientation E[orientation] is color‐coded for orientation according to $\left[\text{red, green, blue}\right]\;\;=\;\;\left[E\left[D_{\mathrm{xx}}\right],E\left[D_{\mathrm{yy}}\right],E\left[D_{\mathrm{zz}}\right]\right]/\text{max}\left(E\left[D_{\mathrm{xx}}\right],E\left[D_{\mathrm{yy}}\right],\;E\left[D_{\mathrm{zz}}\right]\right)$, where the average diffusivities $E[D_{\mathrm{ii}}]$ are associated with the directions i=x,y,z corresponding to the “left‐right,” “anterior‐posterior,” and “superior‐inferior” directions, respectively. For a given bin, the intensity of the bin‐specific maps equals the voxel‐wise average fraction $f_{\mathrm{bin}}$ of components belonging to this bin. Finally, panel (D) contains the global means, variances, and covariances of $D_{\mathrm{iso}}$, $D_{\Delta }^{2}$, and $R_{1}$

**FIGURE 4:**
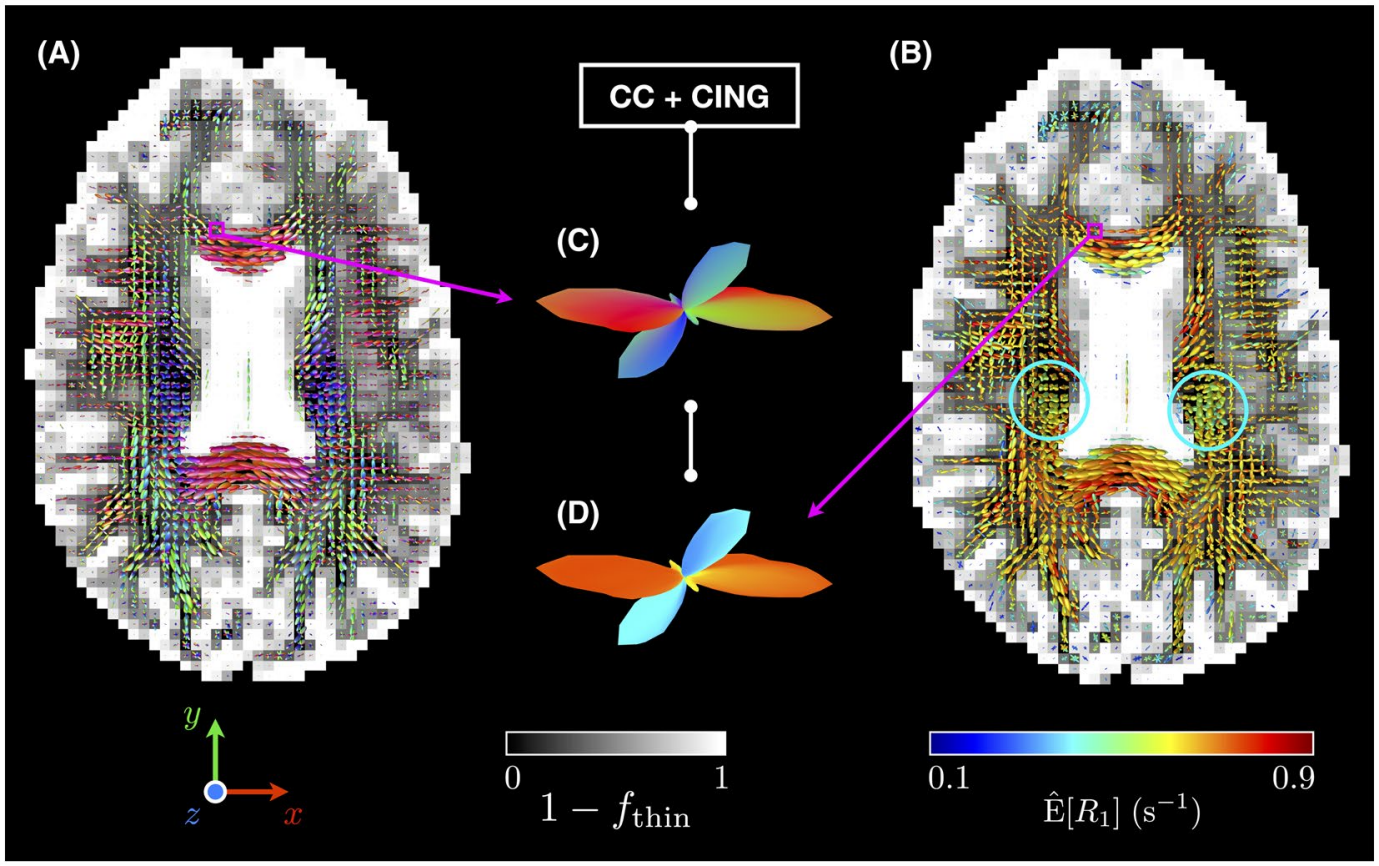
Axial gray‐scale maps of the fraction of non thin‐bin components $1‐f_{\mathrm{thin}}$ with superimposed ODFs colored by (A) local orientation (with x, y and z corresponding to the “left‐right,” “anterior‐posterior,” and “superior‐inferior” directions, respectively) and by (B) $\hat{E}\left[R_{1}\right]$ (see Section 2.3). The middle insets zoom on a voxel containing a fiber crossing between the corpus callosum (CC) and the cingulum (CING), and presents the estimated (C) orientation‐colored and (D) $\hat{E}\left[R_{1}\right]$‐colored ODFs for this voxel. While differences in $\hat{E}\left[R_{1}\right]$ seem to exist between CC and CING, such differences may also exist in the regions where the CST’s pyramidal tracts are located (blue circles), as indicated by the greener $\hat{E}\left[R_{1}\right]$‐colored ODFs therein

**FIGURE 5:**
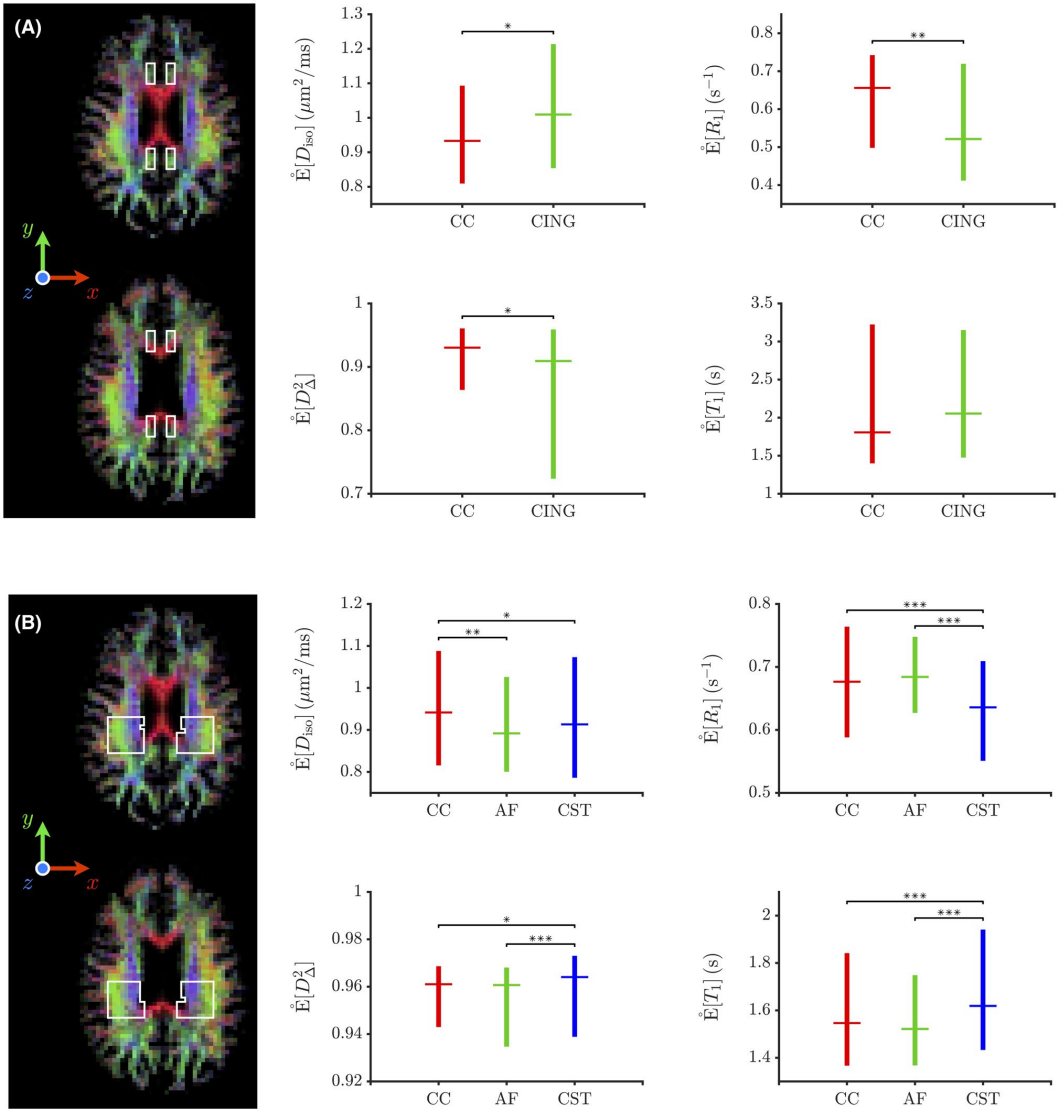
Boxplots of the medians of the orientation‐resolved means $\overset{{}^{\circ}}{E}[\chi ]$ obtained from MC‐DPC (see Section 2.3) within hand‐drawn regions of interest (ROIs), represented as white‐lined boxes over axial slices of the orientation‐colored average fraction of thin‐bin components $f_{\mathrm{thin}}$. For a given boxplot, the horizontal line and whiskers indicate the median and the range between the first and third quartiles of the medians of the orientation‐resolved means $\overset{{}^{\circ}}{E}[\chi ]$, respectively. While panel A’s ROIs focus on crossing areas between the corpus callosum (CC) and the cingulum (CING), those of panel B focus on crossing areas between the corpus callosum (CC), the arcuate fasciculus (AF) and the corticospinal tract (CST) in the posterior corona radiata. Each MC‐DPC cluster (and associated orientation‐resolved means) is robustly assigned to one of these bundles depending on whether its median orientation is closer to the x “left‐right” direction (CC), to the y “anterior‐posterior” direction (CING or AF), or to the z “superior‐inferior” direction (CST). The asterisks report the results of nonparametric Mann‐Whitney U‐tests assessing whether or not two orientation‐resolved means $\overset{{}^{\circ}}{E}[\chi ]$ assigned to distinct bundles are sampled from identically shaped non‐median‐shifted continuous distributions (null hypothesis $H_{0}$). The p‐values resulting from these tests inform on the acceptance or rejection of $H_{0}$ at a certain significance level: 0.05≤p<0.1 (*), 0.01≤p<0.05 (**) and p<0.01 (***)

Figure 2 presents the fitted signals and distributions $P(D,R_{1})$ estimated by our Monte Carlo inversion algorithm in typical voxels associated with WM in the corpus callosum (CC), cortical GM, and CSF in the ventricles. Figure 3 displays typical axial maps of $P(D,R_{1})$’s global and bin‐specific statistical descriptors. Figure 4 shows orientation‐colored and $\hat{E}\left[R_{1}\right]$‐colored ODFs in a typical axial slice. Figure 5 investigates possible microstructural differences between subvoxel fiber populations by leveraging MC‐DPC in regions of interest that target specific fiber crossings, namely the crossing between the CC and the cingulum (CING), and the crossing between the corpus callosum, the arcuate fasciculus (AF), and the corticospinal tract (CST) in the posterior corona radiata.

## 4 DISCUSSION

As observed in previous works,^13^, ^66^, ^95^ Figure 2 demonstrates that the Monte Carlo inversion algorithm yields distributions consistent with the features of measured raw signals in various voxels pertaining to WM, cortical GM, and CSF in the ventricles. In particular, the diffusion isotropy of GM and CSF implies $b_{\Delta }$‐ and b‐tensor orientation‐independent measured signals, and the $\tau_{R}$‐dependence of the $b\;=\;0.1\;\text{ms}/\mu m^{2}$ signals shows that $R_{1}$ increases when going from CSF to GM, and from GM to WM. In addition, the three bins capture these distinct environments in accordance with their original design.

Figure 3 shows that the Monte Carlo inversion algorithm can estimate maps of $P(D,R_{1})$’s statistical descriptors. In particular, it retrieves non‐$R_{1}$‐related maps that are consistent with those thoroughly discussed in Ref. [13]. Let us thus mainly discuss the $R_{1}$‐related maps. The bin‐specific $E[R_{1}]$ maps of Figure 3C present a clear contrast between our tissue‐specific bins, due to high‐$R_{1}$ WM, intermediate‐$R_{1}$ GM, and low‐$R_{1}$ CSF. In Figure 3D, the global $E[R_{1}]$ map resembles an expected low‐resolution conventional $R_{1}$ map, that is, bright in WM, slightly darker in GM, and very dark in CSF. The $V[R_{1}]$ map resembles a noisier version of the $V[D_{\mathrm{iso}}]$ map, as both give high values in mixed CSF‐WM/GM voxels. The noise in $V[R_{1}]$ could be reduced by adding more repetition times in the acquisition scheme. The $C[D_{\mathrm{iso}},R_{1}]$ map is negative at the interface between CSF and either WM or cortical GM. Indeed, upon entering CSF from WM/GM, $D_{\mathrm{iso}}$ increases and $R_{1}$ decreases rapidly. Finally, the $C[D_{\Delta }^{2},R_{1}]$ map exhibits no specific pattern. We emphasize the fact that although $D_{\mathrm{iso}}$ and $R_{1}$ are often correlated in the brain, they nevertheless report on different properties, namely microstructure and chemical composition. Therefore, it may still be useful to map them separately (means and variances) and jointly (covariance), especially in pathological cases.

Figure 4 features nonparametric ODFs capturing local orientations that are consistent with the known anatomy. Regarding $\hat{E}\left[R_{1}\right]$‐colored ODFs (see Section 2.3), they change colors when approaching tissue interfaces with CSF. This gradual change in $\hat{E}\left[R_{1}\right]$ may originate from molecular exchange and/or magnetization transfer between tissues and CSF. Importantly, Figure 4 shows that potential differences in $T_{1}$ relaxation may exist between major fiber bundles, namely CC and CING (insets in Figure 4), and CC, AF, and CST (greener $\hat{E}\left[R_{1}\right]$‐colored ODFs in the CST’s pyramidal tracts).

These potential microstructural differences are quantified in Figure 5. Focusing on relaxation‐based differences, Figure 5A shows that CC and CING exhibit significant differences in $\overset{{}^{\circ}}{E}[R_{1}]$ that are qualitatively consistent with those found in Refs. [8, 33], that is, $R_{1}$ tends to be lower in CING compared to CC. As for Figure 5B, it shows that CST features significant differences in $\overset{{}^{\circ}}{E}[R_{1}]$ and $\overset{{}^{\circ}}{E}[T_{1}]$ with CC and AF, with no statistically significant differences between CC and AF. These differences are qualitatively consistent with those identified for CST in Ref. [8], that is, $T_{1}$ tends to be higher in CST compared to CC and AF. These differences justify the need for a *5D* inversion of the drMRI signal. Indeed, while a 3D inversion of the powder‐averaged drMRI signal is possible, it would only be useful if $R_{1}$ were independent of orientation, which is often not the case.^8^, ^33^

Quantitatively, the $T_{1}$ values estimated by $\overset{{}^{\circ}}{E}[T_{1}]$ in Figure 5 (around 1.5‐2 seconds) are overestimated compared to those of Ref. [8] (around 0.9‐1 second) and Ref. [33] (around 0.7 second). This discrepancy can be explained by the following factors. First, the acquisition scheme described in Section 2. 1 does not maximize the amount of diffusion‐relaxation correlations built into the inversion kernel of Equation (1), because the same diffusion‐weighting block was repeated for each acquired repetition time. Similar problems have been suggested to lead to a loss of accuracy for the Monte Carlo inversion.^36^ Besides, the presence of a similar $T_{1}$ overestimation at SNR = 40 in the in silico evaluation of MC‐DPC presented in Supporting Information Figure S2 indicates that our acquisition sampling scheme is a limiting factor of this present work. Second, the use of saturation recovery with a spoiled spin echo for $T_{1}$ encoding is very sensitive to flip‐angle inaccuracies caused by both $B_{1}^{+}$ inhomogeneity across the subject and slice‐profile imperfections. Saturation‐recovery based $T_{1}$ mapping is also sensitive to magnetization‐transfer effects, especially in the present setup comprising an additional refocusing pulse and a fat‐saturation pulse.^100^, ^101^, ^102^ These technical limitations should be mitigated upon developing a sequence that includes inversion preparation for enhanced $T_{1}$ sensitivity and slice shuffling for optimized time efficiency.^9^, ^10^

## 5 CONCLUSIONS

Diffusion‐$T_{1}$ weighted datasets incorporating multiple b‐tensor shapes can be inverted without relying on limiting assumptions to obtain nonparametric distributions $P(D,R_{1})$ using the Monte Carlo inversion algorithm. The main features of the retrieved distributions can be visualized as maps of global and bin‐specific statistical descriptors related to means, variances, and covariances of diffusion‐relaxation properties. In particular, the bin‐specific $E[R_{1}]$ maps exhibit the expected $R_{1}$ contrast between WM, GM, and CSF. Further insight into WM microstructure is provided by the “thin bin,” which isolates highly anisotropic components that should report on WM tissues. From these thin‐bin components, visualization of fiber‐specific information is improved upon defining ODFs that can be color‐mapped with respect to local orientation or diffusion‐relaxation features.^75^ While $\hat{E}\left[R_{1}\right]$‐colored ODFs hint at possible differences between fiber bundles, MC‐DPC enables their quantification in terms of fiber‐specific diffusion‐relaxation measures.^77^

Importantly, significant relaxation‐based differences are detected between the CC and the CING, and between the CST and the CC and AF. These differences, qualitatively consistent with those found in previous works,^8^, ^33^ offer a first proof of concept for the potential of our Monte Carlo framework in terms of nonparametric fiber‐specific $T_{1}$ relaxometry. Such approach would be practical in identifying differences in $T_{1}$ between distinct subvoxel fiber populations, characterizing developmental or pathological changes in $T_{1}$ within a given subvoxel fiber population, and measuring the angular dependence of longitudinal relaxation times in WM with respect to the main MRI magnetic field $B_{0}$.^103^, ^104^ Moreover, fiber‐specific $T_{1}$ values could be relevant for microstructure‐informed tractography.^105^, ^106^, ^107^

Nevertheless, we emphasize that this work can be improved in multiple ways. First, our acquisition setup could be optimized in terms of speed^9^, ^10^ and sensitivity.^89^, ^108^, ^109^, ^110^ Second, our manual binning could be made data‐driven upon using automatic clustering techniques similar to that of Ref. [77], or upon extending recent works to higher dimensions.^46^, ^98^ Third, better matching between the output of our Monte Carlo framework and plausible WM tracts would be obtained by integrating tractography^111^, ^112^, ^113^, ^114^, ^115^, ^116^, ^117^, ^118^ into our analysis pipeline. Finally, our framework remains to be applied to more subjects to assess its consistency across samples and studies.

## CONFLICT OF INTEREST

D. Topgaard owns shares in Random Walk Imaging AB (Lund, Sweden, http://www.rwi.se/), holding patents related to the described methods.

## Supporting information


**FIGURE S1** Subvoxel orientations retrieved for the in silico data described in Section S3 using the Monte Carlo inversion for various numbers $N_{b}$ of bootstrap solutions and various SNR levels. While the ODFs were obtained via the process detailed in Section S1, the orientational clusters, here represented on the unit sphere, were extracted *via* MC‐DPC according to Section S2. $\overset{{}^{\circ}}{\Delta }\beta$ denotes the angular deviation, computed for a given orientational cluster as the shortest angle between either the cluster geometric median orientation (circles, see Equation S7) or the corresponding ODF peak (squares, see Section S1), and the closest ground‐truth anisotropic component orientation. The color mapped onto the ODF codes for local orientation according to [red, green, blue] ≡ [|*x*|, |*y*|, |*z*|]/max([|*x*|, |*y*|, |*z*|]). As for the clusters, while opacity codes for the weight of the intra‐cluster averaged components (see Equation S6), color codes for the geometric median orientation of each cluster (see Equation S7). The conditions of the in vivo study presented in the main body of the paper are closest to the case $\left(N_{b}\;=\;100,\;\text{SNR}\;=\;40\right)$ **FIGURE S2** Orientation‐resolved means $\overset{{}^{\circ}}{E}$[*χ*] (see Equation S5) and weights $\overset{{}^{\circ}}{w}$ (see Equation S6) associated with the MC‐DPC clusters of Figure S1. While ground truth is shown as horizontal lines, the circles and whiskers represent the medians and interquartile ranges of the orientation‐resolved means across bootstrap solutions, respectively. Squares correspond to the estimated ODF‐peak metrics. Colors match those of the orientational clusters/ODF peaks presented in Figure S1. In the rightmost panels, cluster weights $\overset{{}^{\circ}}{w}$ were normalized so that the sum of all median weights across clusters equals one. Their ODF‐peak equivalents were simply obtained by taking the mesh‐projected component weights (ie, ODF radii) along the peaks of a given ODF (see Section S1). These ODF‐peak weights were then normalized to sum up to one, for easier comparison with normalized cluster weights. The conditions of the in vivo study presented in the main body of the paper are closest to the case $\left(N_{b}\;\;=\;\;100,\;\text{SNR}\;=\;40\right)$

## Data Availability

The code and in silico data that support the findings of this study are openly available in a GitHub repository at https://github.com/areymbaut (last accessed: 22nd of November 2020).
