# Supplementary material for: Toward nonparametric diffusion‐T1 characterization of crossing fibers in the human brain
Source: Magn Reson Med. 2020 Dec 10;85(5):2815–27. doi: 10.1002/mrm.28604 (PMC7898694; doi:10.1002/mrm.28604)
Supplement: Supplementary file 1 — FIGURE S1 Subvoxel orientations retrieved for the in silico data described in Section S3 using the Monte Carlo inversion for various numbers Nb of bootstrap solutions and various SNR levels. While the ODFs were obtained via the process detailed in Section S1, the orientational clusters, here represented on the unit sphere, were extracted via MC‐DPC according to Section S2. Δ°β denotes the angular deviation, computed for a given orientational cluster as the shortest angle between either the cluster geometric median orientation (circles, see Equation S7) or the corresponding ODF peak (squares, see Section S1), and the closest ground‐truth anisotropic component orientation. The color mapped onto the ODF codes for local orientation according to [red, green, blue] ≡ [|x|, |y|, |z|]/max([|x|, |y|, |z|]). As for the clusters, while opacity codes for the weight of the intra‐cluster averaged components (see Equation S6), color codes for the geometric median orientation of each cluster (see Equation S7). The conditions of the in vivo study presented in the main body of the paper are closest to the case (Nb=100,SNR=40) FIGURE S2 Orientation‐resolved means E°[χ] (see Equation S5) and weights w° (see Equation S6) associated with the MC‐DPC clusters of Figure S1. While ground truth is shown as horizontal lines, the circles and whiskers represent the medians and interquartile ranges of the orientation‐resolved means across bootstrap solutions, respectively. Squares correspond to the estimated ODF‐peak metrics. Colors match those of the orientational clusters/ODF peaks presented in Figure S1. In the rightmost panels, cluster weights w° were normalized so that the sum of all median weights across clusters equals one. Their ODF‐peak equivalents were simply obtained by taking the mesh‐projected component weights (ie, ODF radii) along the peaks of a given ODF (see Section S1). These ODF‐peak weights were then normalized to sum up to one, for easier comparison with normalized cluster we [file MRM-85-2815-s001.pdf]

SUPPORTING INFORMATION

This supporting information for "Towards non-parametric diffusion-T_1 characterization of crossing fibers in the human brain" first details the inner workings of our orientation distribution functions (ODFs) [1,2] and Monte-Carlo density-peak clustering (MC-DPC) [3] in Sections S1 and S2, respectively. It then describes the *in silico* data used to perform MC-DPC's *in silico* evaluation in Section S3 and finally discusses this evaluation in Section S4.

S1 | ORIENTATION DISTRIBUTION FUNCTIONS

For each bootstrap solution n_b (with $1 \leq n_\mathrm{b} \leq N_\mathrm{b} = 96$), we considered the voxel-wise discrete ensemble of components belonging to the thin bin,

$$\mathcal{E}^{\mathrm{thin}}_{n_\mathrm{b}} = \{(D_{\|,i}, D_{\perp,i}, \theta_i, \phi_i, R_{1,i}, w_i)\}_{n_\mathrm{b},\ i\in\{\text{thin bin}\}} \,, \tag{S1}$$

and computed an ODF $P_{n_\mathrm{b}}(\theta_\text{mesh}, \phi_\text{mesh})$ on a dense spherical mesh $\{(\theta_\text{mesh}, \phi_\text{mesh})\}$ by convolving the discrete set of components of Equation S1 with a Watson distribution kernel [4,5] as

$$P_{n_\mathrm{b}}(\theta_\text{mesh}, \phi_\text{mesh}) = \sum_{i\in\mathcal{E}^{\mathrm{thin}}_{n_\mathrm{b}}} w_i\, \exp(\kappa\, [\mathbf{u}_i \cdot \boldsymbol{\mu}(\theta_\text{mesh}, \phi_\text{mesh})]^2) \,, \tag{S2}$$

where $\mathbf{u}_i \equiv (\theta_\mathrm{i}, \phi_\mathrm{i})$ is the unit vector giving the orientation of component i, $\boldsymbol{\mu}(\theta_\text{mesh}, \phi_\text{mesh}) \equiv (\theta_\text{mesh}, \phi_\text{mesh})$ is the unit vector associated with a spherical-mesh point, "$\cdot$" denotes the scalar product, and κ is the concentration parameter that regulates the amount of orientation dispersion around $\mathbf{u}_i$ in the Watson kernel. A final voxel-wise ODF $P(\theta_\text{mesh}, \phi_\text{mesh})$ was calculated as the median of the per-bootstrap ODFs:

$$P(\theta_\text{mesh}, \phi_\text{mesh}) = \mathrm{Med}_{(n_\mathrm{b})}\left(P_{n_\mathrm{b}}(\theta_\text{mesh}, \phi_\text{mesh})\right) \,. \tag{S3}$$

The purpose of the Watson kernel is to smoothly map the discrete set of components onto the nearest nodes of the spherical mesh $\{(\theta_\text{mesh}, \phi_\text{mesh})\}$ without inducing any significant peak broadening larger than the distance between two nearest-neighboring mesh nodes. In this work, we considered a 1000-point uniform spherical mesh and set the concentration parameter of the Watson kernel to $\kappa = 14.9$, corresponding in the small-angle limit to a "spherical Gaussian" with standard deviation equal to $1/\sqrt{2\kappa} = 10.5°$. Finally, the mesh-projected $D_{\mathrm{iso},i} = (D_{\|,i} + 2D_{\perp,i})/3$, $D^2_{\Delta,i} = [(D_{\|,i} - D_{\perp,i})/(D_{\|,i} + 2D_{\perp,i})]^2$ and $R_{1,i}$ values enable the computation of orientation-specific diffusion-relaxation means: [1,2]

$$\mathrm{Med}_{(n_\mathrm{b})}\left(\hat{\mathrm{E}}[\chi]_{n_\mathrm{b}}(\theta_\text{mesh}, \phi_\text{mesh})\right) = \mathrm{Med}_{(n_\mathrm{b})}\left(\frac{1}{P_{n_\mathrm{b}}(\theta_\text{mesh}, \phi_\text{mesh})} \sum_{i\in\mathcal{E}^{\mathrm{thin}}_{n_\mathrm{b}}} w_i\, \chi_i\, \exp(\kappa\, [\mathbf{u}_i \cdot \boldsymbol{\mu}(\theta_\text{mesh}, \phi_\text{mesh})]^2)\right) \,, \tag{S4}$$

with $\chi \equiv D_\mathrm{iso}, D^2_\Delta, R_1$. After isolating the peak orientations $\{(\theta_\text{peak}, \phi_\text{peak})\}$ where the median ODF $P(\theta_\text{mesh}, \phi_\text{mesh})$ is locally maximized, ODF-peak measures of $\chi \equiv D_\mathrm{iso}, D^2_\Delta, R_1$ can be obtained by computing Equation S4 at any given peak orientation.

S2 | MONTE-CARLO DENSITY-PEAK CLUSTERING

Let us review the steps through which MC-DPC estimates fiber-specific properties from the output of the Monte-Carlo inversion algorithm. First, MC-DPC gathers the ensemble $\{\mathcal{E}^{\mathrm{thin}}_{n_\mathrm{b}}\}_{1\leq n_\mathrm{b}\leq N_\mathrm{b}}$ of all per-bootstrap thin-bin solution sets $\mathcal{E}^{\mathrm{thin}}_{n_\mathrm{b}}$ (see Equation S1) and delineates N_c clusters in its orientation subspace using DPC with data-point density and outlier detection altered to account for the weights w_i of the retrieved thin-bin components. [3] An initial number of clusters N_c is automatically set by the number of voxel-wise ODF peaks output by our Monte-Carlo framework, [1,2] but may be reduced during the MC-DPC procedure following a filtering approach detailed in Ref. [3]. Second, MC-DPC computes orientation-resolved statistics across bootstrap solutions by separately classifying each per-bootstrap ensemble of thin-bin solutions $\mathcal{E}^{\mathrm{thin}}_{n_\mathrm{b}}$ into N_c ensembles $\mathcal{E}^{\mathrm{thin}}_{n_\mathrm{b},n_\mathrm{c}}$ (with $1 \leq n_\mathrm{c} \leq N_\mathrm{c}$), each containing the thin-bin solutions of bootstrap solution n_b that belong to an estimated cluster n_c. It then averages the properties of the solutions within each ensemble $\mathcal{E}^{\mathrm{thin}}_{n_\mathrm{b},n_\mathrm{c}}$ independently, yielding the orientation-resolved means

$$\mathring{\mathrm{E}}[\chi]_{n_\mathrm{b},n_\mathrm{c}} = \frac{\sum_{k\in\mathcal{E}^{\mathrm{thin}}_{n_\mathrm{b},n_\mathrm{c}}} w_k\, \chi_k}{\sum_{k\in\mathcal{E}^{\mathrm{thin}}_{n_\mathrm{b},n_\mathrm{c}}} w_k} \,, \tag{S5}$$

with $\chi \equiv x, y, z, D_\mathrm{iso}, D^2_\Delta, R_1, T_1 = 1/R_1$, where (x, y, z) are the Cartesian coordinates associated with a component orientation (θ, ϕ). The total weight associated with each orientation-resolved mean is given by

$$\mathring{w}_{n_\mathrm{b},n_\mathrm{c}} = \sum_{k\in\mathcal{E}^{\mathrm{thin}}_{n_\mathrm{b},n_\mathrm{c}}} w_k \,. \tag{S6}$$

The short-hand notations "$\mathring{\hat{\mathrm{E}}}[\chi]$" and "$\mathring{\hat{w}}$" are now used for simplicity to describe the collection of orientation-resolved means $\mathring{\hat{\mathrm{E}}}[\chi]_{n_\mathrm{b},n_\mathrm{c}}$ and weights $\mathring{\hat{w}}_{n_\mathrm{b},n_\mathrm{c}}$ originating from all bootstrap solutions n_b and all clusters n_c. Finally, one can extract the median and interquartile range of the orientation-resolved means $\mathring{\hat{\mathrm{E}}}[\chi]$ and weights $\mathring{\hat{w}}$ across bootstrap solutions.

From a pure orientation standpoint, an equivalent of a median cluster orientation can be computed as the geometric median orientation $\mathbf{u}_{\mathrm{Med},n_\mathrm{c}} \equiv (\theta_{\mathrm{Med},n_\mathrm{c}}, \phi_{\mathrm{Med},n_\mathrm{c}})$ of each cluster-specific collection of mean orientations, $\{\mathring{\hat{\mathrm{E}}}[\mathbf{u}]_{n_\mathrm{b},n_\mathrm{c}}\}_{1\leq n_\mathrm{b}\leq N_\mathrm{b}}$, *via* the argument minimization of the following function dependent on arbitrary unit orientations $\mathbf{v}$:

$$\mathbf{u}_{\mathrm{Med},n_\mathrm{c}} = \underset{\mathbf{v}}{\mathrm{argmin}} \left[\sum_{N_\mathrm{b}=1}^{N_\mathrm{b}} \mathring{\hat{w}}_{N_\mathrm{b},n_\mathrm{c}}\, d(\mathbf{v}, \mathring{\hat{\mathrm{E}}}[\mathbf{u}]_{N_\mathrm{b},n_\mathrm{c}}) \right], \tag{S7}$$

using the antipodally symmetric angular distance

$$d(\mathbf{u}_i, \mathbf{u}_j) = \arccos(|\cos\theta_i \cos\theta_j + \sin\theta_i \sin\theta_j \cos(\phi_i - \phi_j)|)\,, \tag{S8}$$

with $\mathbf{u}_i \equiv (\theta_i, \phi_i)$ and $\mathbf{u}_j \equiv (\theta_j, \phi_j)$.

S3 | NUMERICAL DATA

An *in silico* evaluation of MC-DPC was performed by designing a set of components $(D_{\|,i}, D_{\perp,i}, \theta_i, \phi_i, R_{1,i}, w_i)$ mimicking a three-way crossing and an isotropic component to simulate partial voluming. Acknowledging that MC-DPC quantifies the median value and uncertainty of *average* fiber-specific diffusion-relaxation features, a given *in silico* "fiber" was defined as the combination of three Gaussian distributions, *i.e.* one in the isotropic-diffusivity dimension, one in the normalized-anisotropy dimension and one in the longitudinal-relaxation dimension, with respective means $\overline{D_\mathrm{iso}}$, $\overline{D_\Delta}$ and $\overline{T_1}$, and a constant standard-deviation-to-mean ratio of 0.02. The distributions of θ and ϕ values were set as Dirac-delta distributions, for simplicity. Orders of magnitude for $\overline{D_\mathrm{iso}}$, $\overline{D_\Delta}$ and $\overline{T_1}$ were inspired by Ref. [6], giving the following compartments:

- one isotropic component, $D_\mathrm{iso} = 2$ μm²/ms, $T_1 = 4$ s, $w_\mathrm{iso} = 0.1$.
- one fiber along x $(\theta = \pi/2, \phi = 0)$, total weight $= (1 - w_\mathrm{iso}) \times 0.26$, $\overline{D_\mathrm{iso}} = 0.75$ μm²/ms, $\overline{D_\Delta} = 0.95$, $\overline{T_1} = 0.8$ s.
- one fiber along y-z $(\theta = \pi/4, \phi = \pi/2)$, total weight $= (1 - w_\mathrm{iso}) \times 0.33$, $\overline{D_\mathrm{iso}} = 0.8$ μm²/ms, $\overline{D_\Delta} = 0.9$, $\overline{T_1} = 1$ s.
- one fiber along z $(\theta = 0, \phi = 0)$, total weight $= (1 - w_\mathrm{iso}) \times 0.41$, $\overline{D_\mathrm{iso}} = 0.85$ μm²/ms, $\overline{D_\Delta} = 0.85$, $\overline{T_1} = 1.2$ s.

The ground-truth signals associated with these systems were computed using the same inversion kernel and acquisition scheme as those detailed in the main body of this paper. This calculation is in agreement with the conventional procedure for testing 1D, 2D or 4D Laplace inversion algorithms, where the ground-truth signal is calculated with the same kernel as the inversion.[7–11] Rician noise was then added to the ground-truth signals according to

$$\mathcal{S} = \sqrt{\left(\mathcal{S}_\mathrm{gt} + \frac{\nu}{\mathrm{SNR}}\right)^2 + \left(\frac{\nu'}{\mathrm{SNR}}\right)^2}\,, \tag{S9}$$

where $\mathcal{S}_\mathrm{gt}$ is a ground-truth signal, $\mathcal{S}$ is the corresponding noisy signal, ν and ν' denote random numbers drawn from a normal distribution with zero mean and unit standard deviation, and SNR is the desired signal-to-noise ratio. Three SNRs were considered: the $\mathrm{SNR} = 40$ of our *in vivo* dataset, and higher SNR values, namely $\mathrm{SNR} = 70$ and $\mathrm{SNR} = 90$. For each of the aforementioned SNRs, the signals obtained from Equation S9 were inverted using the 5D Monte-Carlo inversion detailed in the main body of the paper with either $N_\mathrm{b} = 50$, $N_\mathrm{b} = 75$ or $N_\mathrm{b} = 100$ bootstrap solutions. Indeed, varying N_b may be relevant when validating MC-DPC because the number of data points that serve as its input is roughly proportional to N_b.[3] Finally, MC-DPC was run on the solutions retrieved from each of these inversions and noise levels to extract orientation-resolved means and weights (see Equations S5 and S6).

S4 | NUMERICAL EVALUATION

Figure S1 displays the sub-voxel orientations retrieved for the *in silico* data described in Section S3 using both the ODFs of Section S1 and MC-DPC from Section S2, and quantifies the angular deviation $\mathring{\Delta}\beta$ of each cluster geometric median orientation (see Equation S7) compared to the closest

ground-truth fiber orientation. Figure S2 quantifies the orientation-resolved means $\mathring{\mathrm{E}}[\chi]$ (see Equation S5) for the same *in silico* data. ODF-peak information is also displayed on Figures S1 and S2 for comparison.

Within Figure S1, the orientational spread of the MC-DPC clusters informs on the precision of the underlying Monte-Carlo signal inversion algorithm. Clusters become more orientationally dispersed as SNR decreases at constant N_b, which is associated with the loss of precision of the 5D Monte-Carlo inversion with reduced SNR. At constant SNR, the orientational clusters retrieved from MC-DPC are rather unaffected upon changing N_b. As for the angular deviation $\mathring{\Delta}\beta$, informing on the accuracy of the underlying Monte-Carlo signal inversion algorithm, it increases with decreasing SNR, especially at $\mathrm{SNR} = 40$. Compared to ODF-peak orientations, cluster geometric median orientations seem to be more accurate as they typically yield smaller values of $\mathring{\Delta}\beta$, probably due to the fact that MC-DPC clusters are not bound to a discrete spherical mesh. However, ODF-peak orientations appear to be more resilient to noise.

Regarding Figure S2, the estimations of the orientation-resolved means and weights tend to suffer from the same loss of accuracy and precision mentioned for the orientational information in Figure S1 as SNR decreases at constant N_b. Estimation performances are not affected by N_b within the investigated range of practical N_b values. First, it is worth noting that $\mathring{\mathrm{E}}[D_\Delta^2]$'s estimations appear to always yield a value close to $\mathring{\mathrm{E}}[D_\Delta^2] = 0.8$, which may be due to a flatness of the fitting landscape along this dimension. Nevertheless, the $\mathrm{SNR} = 70$ and $\mathrm{SNR} = 90$ cases appear to yield rather accurate estimations of the changes in $\mathring{\mathrm{E}}[R_1]$ and $\mathring{\mathrm{E}}[T_1]$ across fiber populations while yielding satisfying accuracy for $\mathring{\mathrm{E}}[D_\mathrm{iso}]$. However, the $\mathrm{SNR} = 40$ case, *i.e.* that closest to the *in vivo* study presented in the main body of the paper, seems to poorly estimate $\mathring{\mathrm{E}}[D_\mathrm{iso}]$ and, importantly, $\mathring{\mathrm{E}}[T_1]$ for certain fiber populations. Similar discrepancies are reported for $\mathring{\mathrm{E}}[T_1]$ in the *in vivo* study presented in the main body of the paper. As for ODF-peak metrics, they generally agree with the median orientation-resolved means at all SNR levels.

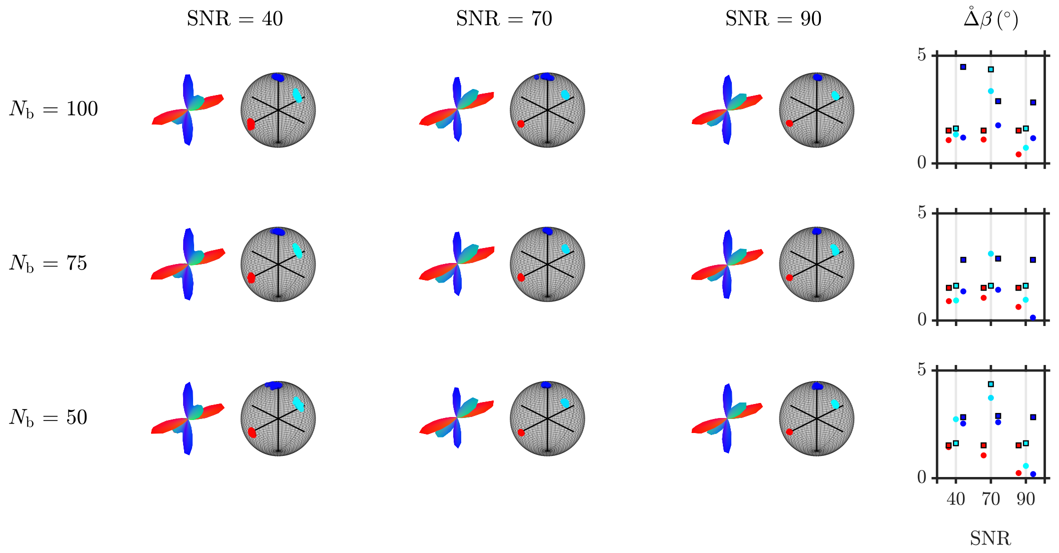

FIGURE S1 Sub-voxel orientations retrieved for the *in silico* data described in Section S3 using the Monte-Carlo inversion for various numbers N_b of bootstrap solutions and various SNR levels. While the ODFs were obtained via the process detailed in Section S1, the orientational clusters, here represented on the unit sphere, were extracted *via* MC-DPC according to Section S2. $\mathring{\Delta}\beta$ denotes the angular deviation, computed for a given orientational cluster as the shortest angle between either the cluster geometric median orientation (circles, see Equation S7) or the corresponding ODF peak (squares, see Section S1), and the closest ground-truth anisotropic component orientation. The color mapped onto the ODF codes for local orientation according to $[\mathrm{red}, \mathrm{green}, \mathrm{blue}] \equiv [|x|, |y|, |z|]/\max([|x|, |y|, |z|])$. As for the clusters, while opacity codes for the weight of the intra-cluster averaged components (see Equation S6), color codes for the geometric median orientation of each cluster (see Equation S7). The conditions of the *in vivo* study presented in the main body of the paper are closest to the case $(N_\mathrm{b} = 100, \mathrm{SNR} = 40)$.

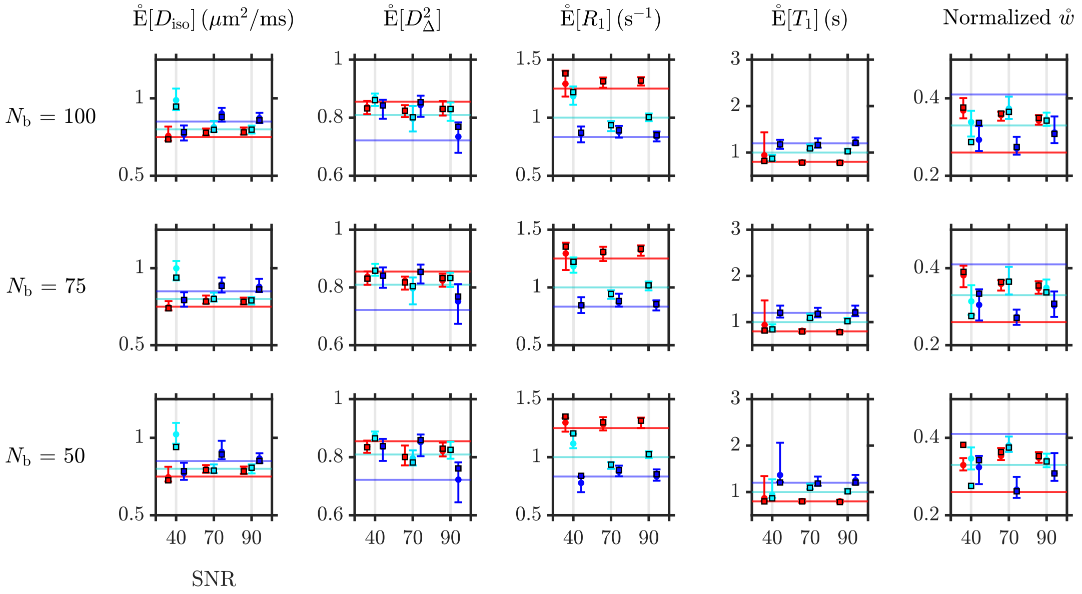

FIGURE S2 Orientation-resolved means $\mathring{\mathrm{E}}[\chi]$ (see Equation S5) and weights $\mathring{w}$ (see Equation S6) associated with the MC-DPC clusters of Figure S1. While ground-truth is shown as horizontal lines, the circles and whiskers represent the medians and interquartile ranges of the orientation-resolved means across bootstrap solutions, respectively. Squares correspond to the estimated ODF-peak metrics. Colors match those of the orientational clusters/ODF peaks presented in Figure S1. In the rightmost panels, cluster weights $\mathring{w}$ were normalized so that the sum of all median weights across clusters equals one. Their ODF-peak equivalents were simply obtained by taking the mesh-projected component weights (*i.e.* ODF radii) along the peaks of a given ODF (see Section S1). These ODF-peak weights were then normalized to sum up to one, for easier comparison with normalized cluster weights. The conditions of the *in vivo* study presented in the main body of the paper are closest to the case (N_b = 100, SNR = 40).

References

1. de Almeida Martins JP, Tax CMW, Reymbaut A, Szczepankiewicz F, Jones DK, Topgaard D. Visualizing orientation-specific relaxation-diffusion features mapped onto orientation distribution functions estimated via nonparametric Monte Carlo MRI signal inversion. *bioRxiv* 2020. doi: 10.1101/2020.05.23.111963

2. de Almeida Martins JP. *Pushing diffusion MRI towards new dimensions*. PhD thesis. Lund University, Lund University, Faculty of Science, Department of Chemistry, Division of Physical Chemistry; 2020.

3. Reymbaut A, de Almeida Martins JP, Tax CMW, Szczepankiewicz F, Jones DK, Topgaard D. Resolving orientation-specific diffusion-relaxation features via Monte-Carlo density-peak clustering in heterogeneous brain tissue. *arXiv e-prints* 2020.

4. Watson GS. Equatorial Distributions on a Sphere. *Biometrika* 1965; 52(1/2): 193–201.

5. Mardia K, Jupp P. *Directional Statistics*. Wiley Series in Probability and StatisticsWiley . 2009.

6. de Santis S, Assaf Y, Jeurissen B, Jones DK, Roebroeck A. T1 relaxometry of crossing fibres in the human brain. *NeuroImage* 2016; 141: 133 - 142. doi: https://doi.org/10.1016/j.neuroimage.2016.07.037

7. Provencher SW. A constrained regularization method for inverting data represented by linear algebraic or integral equations. *Computer Physics Communications* 1982; 27(3): 213 - 227. doi: https://doi.org/10.1016/0010-4655(82)90173-4

8. Whittall KP, MacKay AL. Quantitative interpretation of NMR relaxation data. *Journal of Magnetic Resonance (1969)* 1989; 84(1): 134 - 152. doi: https://doi.org/10.1016/0022-2364(89)90011-5

9. Venkataramanan L, Yi-Qiao Song , Hurlimann MD. Solving Fredholm integrals of the first kind with tensor product structure in 2 and 2.5 dimensions. *IEEE Transactions on Signal Processing* 2002; 50(5): 1017-1026. doi: 10.1109/78.995059

10. Mitchell J, Chandrasekera T, Gladden L. Numerical estimation of relaxation and diffusion distributions in two dimensions. *Progress in Nuclear Magnetic Resonance Spectroscopy* 2012; 62: 34 - 50. doi: https://doi.org/10.1016/j.pnmrs.2011.07.002

11. Reymbaut A, Mezzani P, de Almeida Martins JP, Topgaard D. Accuracy and precision of statistical descriptors obtained from multidimensional diffusion signal inversion algorithms. *NMR in Biomedicine* 2020: e4267. doi: 10.1002/nbm.4267
